# Willingness to Use HIV Pre-exposure Prophylaxis among Community Pharmacy Users in Uganda

**DOI:** 10.21203/rs.3.rs-11230065/v1

**Published:** 2026-10-22

**Authors:** Vivian Nakate, Eva Agnes Laker Odongpiny, Matthew M. Hamill, Annet A. Onzia, Johan H. Melendez, Steven J Reynolds, Barbara Castelnuovo, Rosalind Parkes-Ratanshi, Yukari C. Manabe, Agnes N. Kiragga

## Abstract

HIV Pre-Exposure Prophylaxis (PrEP) uptake is limited in Uganda. Participants were recruited from 18 Kampala community pharmacies (CP); 52.2% had sexually transmitted infection (STI) symptoms, and 62.2% were females. Of the 424 participants with negative or unknown HIV status, only 127 (30.0%) were aware of PrEP; 91.0% reported willingness to use oral and 58.5% injectable PrEP. Preferences for PrEP delivery were at a community pharmacy (44.8%), health facility (48.7%), and home (6.6%). Use of CPs for distribution could increase PrEP uptake.

